# Experimental Evaluation of the Properties of Recycled Aggregate Pavement Brick with a Composite Shaped Phase Change Material

**DOI:** 10.3390/ma15165565

**Published:** 2022-08-13

**Authors:** Chaojie Ru, Guoxin Li, Fanxing Guo, Xuedi Sun, Deliang Yu, Zheng Chen

**Affiliations:** College of Materials Science and Engineering, Xi’an University of Architecture and Technology, Xi’an 710055, China

**Keywords:** construction waste, recycled aggregate, pavement bricks, phase change materials, thermal conductivity

## Abstract

Brick waste makes up a significant part of the solid waste that is generated from building demolition globally. The disposal of this waste consumes land, causes environmental pollution, and is a waste of resources. In order to use this construction waste and increase its functionality, two types of stable-shape PEG-400/SiO_2_ composite shaped PCM and Tet/SiO_2_ composite shaped PCM were studied and added to recycled aggregate pavement bricks, and two new types of composite shaped PCM recycled aggregate pavement bricks were created. SEM, DSC, TGA, and other test methods found the two PCMs to be successfully adsorbed by SiO_2_, and the setting effect of PEG-400/SiO_2_ was found to be better than that of Tet/SiO_2_. The physicochemical properties of both composite shaped PCMs remained stable within the TGA test temperature range. The prepared PCM was added to the recycled aggregate pavement brick. A comprehensive analysis of the properties of the composite shaped PCM recycled orthopedic pavement brick found the compressive strength and flexural strength of Tet/SiO_2_ PCM recycled aggregate pavement brick to be significantly higher than those of PEG-400/SiO_2_ PCM recycled aggregate pavement brick. With a recycled aggregate content of 60% and a compound shaped PCM content of 5%, the 28-day strength of the recycled aggregate pavement brick was found to be higher than that of the recycled aggregate pavement brick with a recycled aggregate content of 70% and a compound shaped PCM content of 10%. This study provides reference for the optimization and upgrading of the thermal storage performance of composite shaped PCM in practical applications, and is of great significance for promoting thermal energy storage development and expanding its application range.

## 1. Introduction

As the global population has increased rapidly, and with the acceleration of urbanization and the development of industry, the problems of energy consumption and environmental pollution have recently gained increasing prominence [1,2]. It has been reported that the European Union and the United States produce approximately 800 million tons and 700 million tons of construction waste each year, respectively [3,4]. China is the largest developing country in the world, and produces approximately 1.8 billion tons of construction waste each year through its building demolition activities [5,6,7]. The demolition of buildings on a global scale makes the utilization rate of the majority of construction waste low, and the utilization rate of renewable energy is relatively low as a result of the influences of the climate, geographical location, and time [8,9]. Construction waste is inevitable in the course of the development of the national economy. A significant amount of construction waste is bad for the limited resources of a country, and it also has a huge impact on the environment. Therefore, effectively utilizing construction waste as a resource; exploring efficient storage and conversion technologies for green, sustainable, and renewable energy; and developing new energy can effectively improve energy utilization. These aspects are of great significance for the realization of energy conservation and environmental protection [10,11].

Phase change materials (PCMs) are a class of functional materials with the capability of storing and releasing heat during the phase change process. They can utilize PCMs to acheive a balance between energy supply and demand [12]. Due to the advantages of high energy storage density, strong energy storage capacity, and constant temperature that it possesses, this material is widely used in energy conservation in buildings [13], road engineering [14], air conditioning cold storage [15], and the utilization of solar energy [16]. As an energy storage material, organic phase change materials have the advantages of needing no supercooling, needing no precipitation, stable performance, low corrosiveness, low price, and strong availability [17,18]. Polyethylene glycol (PEG) is an organic phase change material of polyethers. It is a long-chain polymer that is composed of -CH_2_CH_2_O- with hydroxyl groups at both ends, and it demonstrates good hydrophilicity [12]. When the temperature increases to the phase transition temperature, PEG transforms from an ordered solid-state crystal into a disordered liquid state, demonstrating the typical properties of an organic solid–liquid phase change material [19]. Tetradecane (Tet) is a saturated aliphatic hydrocarbon organic phase change material with the chemical formula C_14_H_30_. In a solid state, its molecular chain conformation slowly changes from a straight chain to a curved state as the temperature increases, whereas in a liquid state, the conformation of the molecular chain slowly changes from a curved state to a straight chain as the temperature increases [20,21]. However, as a result of the low thermal conductivity and low density of organic materials, there are limitations to their application and development. A single PCM does not satisfy the needs of practical applications, and composite PCM is obtained by mixing and combining multiple PCMs using a certain combination method [22]. This effectively combines the properties of a variety of materials, such that composite phase change materials are currently a research direction that has great research potential [23]. Wang et al. [24] prepared a PEG-400/SiO_2_ composite shaped PCM with an inexpensive porous material, SiO_2_, as the carrier and PEG as the core material. The results showed that the actual maximum adsorption amount of PEG is 47.9%, and the obtained composite demonstrates good thermal stability. Following 100 thermal cycles, the phase transition enthalpy loss is small, and the melting point and freezing point temperature remain relatively stable, have good thermal reliability, and are suitable for a building envelope. Chen et al. [25] prepared a composite phase change material with SiO_2_ as the shell and PEG as the PCM, incorporating it into porous asphalt concrete as a replacement for fine aggregate. The effects that the particle size and dosage of PEG-400/SiO_2_ composite phase change material have on the internal temperature of porous asphalt concrete were experimentally studied. It was found that the PEG-400/SiO_2_ composite PCM with a 70% PEG mass fraction is an excellent candidate for porous asphalt concrete. Xu et al. [26] prepared a new type of concrete block with PEG-400/SiO_2_ composite PCM, studying its performance and architectural application potential. They found that the amount of PEG-400/SiO_2_ composite PCM significantly affects the properties of phase change material concrete bricks. When the amount of PCM added is 0–18%, the compressive strength decreases from 23.6 to 0.69 MPa, and the thermal conductivity decreases from 0.94 to 0.59 W/(m·K). The optimum amount of PCM in the concrete block was determined to be 7.5%. Yan et al. [27] used PEG with a molecular weight of 6000 as the PCM, and SiO_2_ containing aminopropyl and carboxyl multi-walled carbon nanotubes as the support material, synthesizing the composite phase change material using the sol-gel method. Fourier transform infrared spectroscopy (FTIR), an X-ray diffractometer (XRD), scanning electron microscopy (SEM), and other testing methods were used, and it was found that the prepared PEG/SAM PCM had high thermal conductivity, high energy storage density, photothermal conversion, high storage efficiency, and a stable shape. This proves that photothermal conversion and phase change energy storage materials have great solar energy application potential. Karaman et al. [28] chose PEG with a molecular weight of 1000 as the PCM and diatomite as the carrier material, preparing a new type of PEG/diatomite composite PCM with a stable morphology using the vacuum impregnation method. The use of FTIR, a differential scanning calorimeter (DSC), and a thermogravimetric analyzer (TGA) proved that PEG/diatomite composites have excellent thermal properties and chemical stability, and it is possible to use them directly without additional packaging. Fang et al. [29] synthesized a novel polystyrene/Tet composite nano-encapsulated PCM through ultrasonic-assisted manumission in-situ polymerization as a potential functional thermal fluid (LFTF) for the low-temperature storage of energy. Test methods including FTIR, DSC, and TGA have shown the composite PCM to have good thermal properties and cold storage potential. Its melting point, freezing point, and latent heat are 4.04 °C, 3.43 °C, 98.71 J/g and 91.27 J/g, respectively. Bai et al. [30] utilized melamine urea formaldehyde resin as the wall material and Tet as the core material for the preparation of microencapsulated PCM with the ability to store energy and release heat at a low temperature through in-situ polymerization, using them as modifiers for the preparation of new asphalt phase change materials. The effects that the different core-to-wall ratios, synthesis temperatures, and emulsification speeds had on the morphology, particle size, and thermal stability of microcapsules were studied. The results showed that when the core–wall ratio was 1:1, the stirring temperature was 75 °C, and the emulsification stirring rate was 3 000 r/min, the microcapsule PCM exhibited the best performance, and the latent heat of phase change achieved 119 J/g. The microcapsule PCM can ensure that the structure remains intact in the phase change asphalt, and does not seriously affect asphalt road performance. Fang et al. [31] used Tet as the core material and urea-formaldehyde resin as the shell material in order to study the preparation and characterization of nano-coated Tet PCM. The results revealed that the as-prepared microcapsule phase change material has an endothermic latent heat storage capacity of 100–130 kJ/kg at 5–9 °C, and Tet is effectively encapsulated with good thermal stability.

The low-temperature phase change material can be utilized for the preparation of snow-melting as well as ice-melting pavement bricks, and has certain application prospects in low-temperature snowfall areas. For the purpose of studying low-temperature phase change materials and their application value in low-temperature snow melting pavement bricks, two new composite-shaped PCMs (PEG-400/SiO_2_ and Tet/SiO_2_) were prepared in this study. These were analyzed by SEM, DSC, TGA, and other testing methods to characterize their microstructure, phase transition temperature, and thermal stability. Based on this, two additional composite-shaped PCM recycled aggregate paving bricks (PEG-400/SiO_2_ and Tet/SiO_2_) were prepared, and their mechanical properties and thermal conductivity were further examined. The research findings of this paper investigate the potential application value of two new phase change materials in snow-melting pavement bricks from a theoretical level, which in turn offers a theoretical reference for the preparation of low-temperature snow-melting pavement bricks.

## 2. Materials and Methods

### 2.1. Raw Materials and Proportion

PEG-400 produced by Tianjin Kemio Chemical Reagent Co., Ltd. (Tianjin, China) and Tet from Shanghai Merrill Chemical Technology Co., Ltd. (Shanghai, China) will be used as the PCM. The imported German Evonik Degussa nano-gas phase silica (SiO_2_) will be used as the carrier material for adsorbing PCM (PEG-400 and Tet), which is liquid at room temperature, as the core material of the microcapsules. Anhydrous ethanol produced by Tianjin Fuyu Fine Chemical Co., Ltd. (Tianjin, China) will be used as the organic solvent, diisooctyl sebacate produced by Sinopharm Chemical Reagent Co., Ltd. (Shanghai, China) will be used as the plasticizer, and ethyl cellulose produced by Tianjin Kemio Chemical Reagent Co., Ltd. (Tianjin, China) will be used as the film-forming material to make the wall materials of the microcapsules. The physical parameters of the capsule core and the capsule wall materials can be seen in Table 1. The proportions of the two composite shaped phase change materials can be seen in Table 2.

Composite shaped PCM recycled aggregate paver is prepared from ordinary portland cement, recycled aggregate, composite shaped PCM, and water. The P·O42.5 ordinary portland cement is produced by Anhui Hailuo Group Co., Ltd., and the recycled aggregate was obtained by crushing and classifying old brick waste after dismantling. The mix of composite shaped PCM recycled aggregate pavement bricks can be seen in Table 3. For example, “J-70-0” refers to the benchmark recycled aggregate pavement brick with 70% recycled aggregate and no PCM, “P-70-5” refers to composite shaped phase change material recycled aggregate pavement brick with 70% recycled aggregate and 5% PEG-400/SiO_2_ composite shaped PCM, and “T-70-5” refers to composite shaped phase change material recycled aggregate pavement brick with 70% recycled aggregate and 5% Tet/SiO_2_ composite shaped PCM.

### 2.2. Preparation of composite shaped PCM

#### 2.2.1. Preparation Principle

PCM is physically adsorbed by SiO_2_ and forms a composite shaped PCM core. The plasticizer (diisooctyl sebacate) is then dissolved in an organic solution and added to the film-forming material (ethyl cellulose) to form a liquid-phase gelling system. SiO_2_ is composed of a large number of spherical particles, which cluster together and form small particles; after it is soaked in ethyl cellulose, diisooctyl sebacate solution and film-forming material, the intermolecular force becomes stronger, the particles increase, and the coating film makes the surface of the particles smooth. The gelling system is then mixed with the core material according to the proportion, and is then wrapped on the surface of the core as the capsule wall material. Finally, the organic solvent is evaporated by being heated, and the gelled organic solvent becomes a three-dimensional network structure film, which forms stable microcapsules around the core material.

#### 2.2.2. Preparation Process

**Figure 1:**
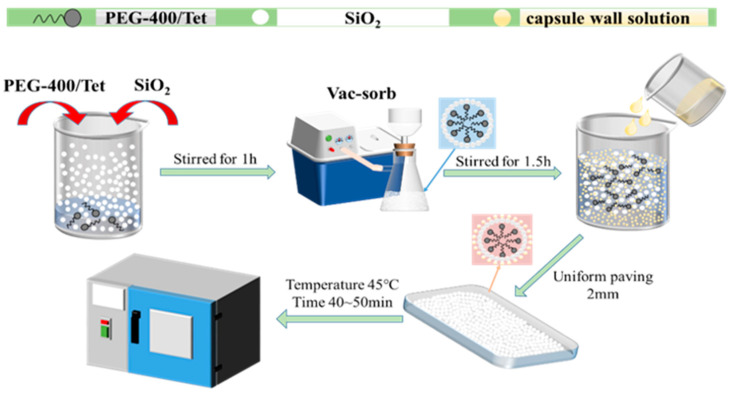
Preparation process for composite shaped PCM.

Firstly, a certain quality of PEG-400/Tet and SiO_2_ are weighed into the beaker and stirred evenly, enabling the SiO_2_ to physically adsorb PEG-400/Tet under normal pressure. It is then moved to the vacuum environment for full adsorption, such that the PCM can be completely adsorbed into the SiO_2_ pores and form the microcapsule core material. Secondly, a certain proportion of absolute ethanol solution is weighed in the beaker as the organic solvent, providing an organic environment for microencapsulated phase change material synthesis. The plasticizer is then slowly and carefully added to the absolute ethanol solution and moved to the magnetic stirrer, where it is stirred evenly. A quantitative amount of ethyl cellulose is then slowly and evenly added to the absolute ethanol and plasticizer mixture, before being continuously stirred until the ethyl cellulose is completely dissolved, enabling the mixture to turn into a pale yellow and transparent gel, which is then taken out to stand. Finally, the products obtained in the first two steps are mixed using the correct proportions, stirred well, and spread thinly in a shallow dish, before being placed into an oven for drying at 45 °C for 50~60 min. The dried products are ground gently, and then sealed and stored; they are sieved to make a composite shaped PCM. The entire experimental process can be seen in Figure 1.

### 2.3. Preparation of Composite Shaped PCM Recycled Aggregate Pavement Bricks

**Figure 2:**
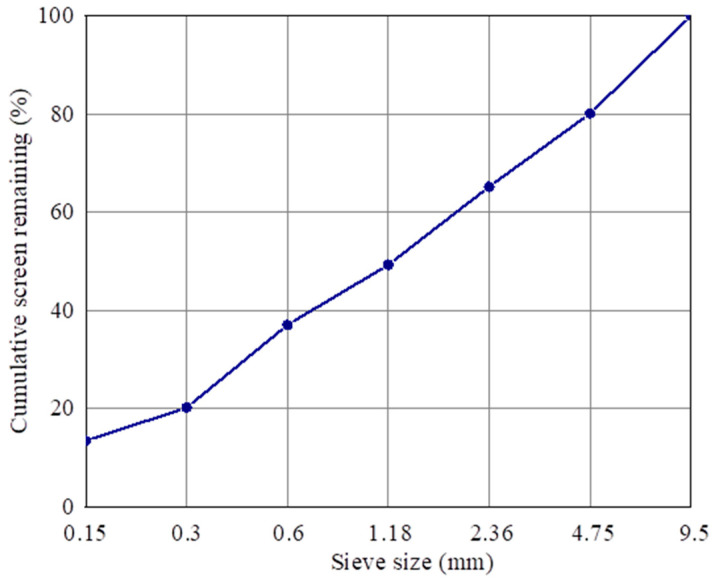
Gradation curve of recycled aggregate particles.

The pavement bricks in this study are made of concrete, the raw materials of which include recycled aggregate, cement, composite shaped PCM, and water. The preparation method is as follows: (1) weigh a certain quantity of recycled aggregate, cement, and composite shaped PCM using the correct mix proportion, place them in a mixing pot, add 60% mixing water, and stir for 1 minute, before adding the remaining mixing water and mixing for an additional 2 min. (2) Add the evenly mixed mixture to the mold twice, and place it on the press for molding following installation. (3) Remove the test block from the mold after it has rested there for 12 h; this is the prepared composite shaped PCM recycled aggregate pavement brick (size 40 mm × 40 mm × 160 mm). (4) Wrap the demoulded pavement bricks with plastic wrap, and then put them into a standard curing room for curing. The temperature of the curing room should be 20 ± 2 °C, and the relative humidity should be above 95%. Figure 2 shows the particle gradation curve of the recycled aggregate in this study.

According to the industry standard of China’s construction industry (construction waste recycled aggregate solid brick, JG/T505-2016, https://www.doc88.com/p-2008634155360.html, accessed on 11 August 2022), recycled aggregate pavement bricks can be divided into six grades according to their compressive strength, as shown in Table 4.

### 2.4. Experimental Methods

The micromorphology of the samples was characterized using SEM (Zeiss sigma-300). A small amount of powder sample was evenly dispersed on the conductive adhesive, and it was sprayed gold before testing as a means of improving the conductivity of the samples.

DSC (American TA q2000) was used to measure the phase transition temperature and phase transition enthalpy of composite shaped PCM and pure PEG/Tet with different mass fractions. It was scanned in a nitrogen atmosphere at a rise/drop rate of 10 °C/min, and the test temperature range was −80~80 °C. The theoretical phase transition enthalpy of the composite shaped PCM was calculated using the following equation [32]:

*∆H_T_* = *∆H_PCMs_* × *ω*, (1)

where *∆H_T_* iss the theoretical phase transition enthalpy of the composite shaped PCM, J/g; *∆H_PCMs_* is the enthalpy of the phase transition of pure PCM, J/g; and *ω* is the mass percentage of PCM in composite shaped PCM, wt.%.

TGA (TA-TGA55) was used to test the thermal stability of PEG-400/SiO_2_ composite shaped PCM and Tet/SiO_2_ composite shaped PCM. The test was conducted in a nitrogen atmosphere at a heating rate of 10 °C/min, and the test temperature was 30~800 °C.

Each composite shaped PCM recycled aggregate pavement brick (size 40 mm × 40 mm × 160 mm) was subjected to a mechanical property test, including testing for compressive strength and flexural strength, after 3, 7, and 28 days. The loading speed of the compressive strength was 2.4 kN/s, and for the flexural test, it was 50 N/s. Three prism samples were used for each composite shaped PCM recycled aggregate pavement brick, and the average value was taken as the strength result.

Different composite shaped PCM recycled aggregate pavement brick heat conduction plates (size 200 mm × 200 mm × 20 mm) were prepared for thermal conductivity testing using the flat plate heat conduction instrument produced by Beijing Zhongke Tianhao Technology Co., Ltd. The heat conducting plate—which had been cured for 28 days—was taken out from the curing room, any surface moisture was wiped away, and it was placed between the hot plate and the cold plate after drying. The temperature of the hot plate was 35 °C, and that of the cold plate was 15 °C.

### 2.5. Temperature Control Range Calculation

In order to analyze the heat storage capacity of the composite shaped PCM recycled pavement bricks, the temperature control effect of the prepared pavement bricks can be evaluated by calculating the theoretical temperature rise and reduction. Generally, the specific heat capacity of concrete is about 900 J/(kg·°C). If the addition amount of phase change material is known, the theoretical temperature rise and reduction range can be calculated using follow equation:

(2) $$∆T=w\frac{H}{c}$$

where ∆*T* is the theoretical temperature rise and reduction range, %; *w* is the mass percentage of the composite shaped PCM in the pavement brick, %; and *c* is the specific heat capacity of the composite shaped PCM pavement brick, J/(kg·°C). Because the recycled aggregate selected in this study was made of waste concrete, the *c* value was taken as 900 J/(kg·°C).

## 3. Results and Discussion

### 3.1. Microstructure

The particle size of the composite shaped PCM that was prepared in this study was approximately 1mm. The material felt dry, there was no liquid leakage, and the particles were distinct. However, it was difficult to determine the specific encapsulation effect purely by examining it with the naked eye. Therefore, the prepared composite shaped PCM was observed using a scanning electron microscope. The appearance and wrapping effect of the material were then analyzed [33].

**Figure 3:**
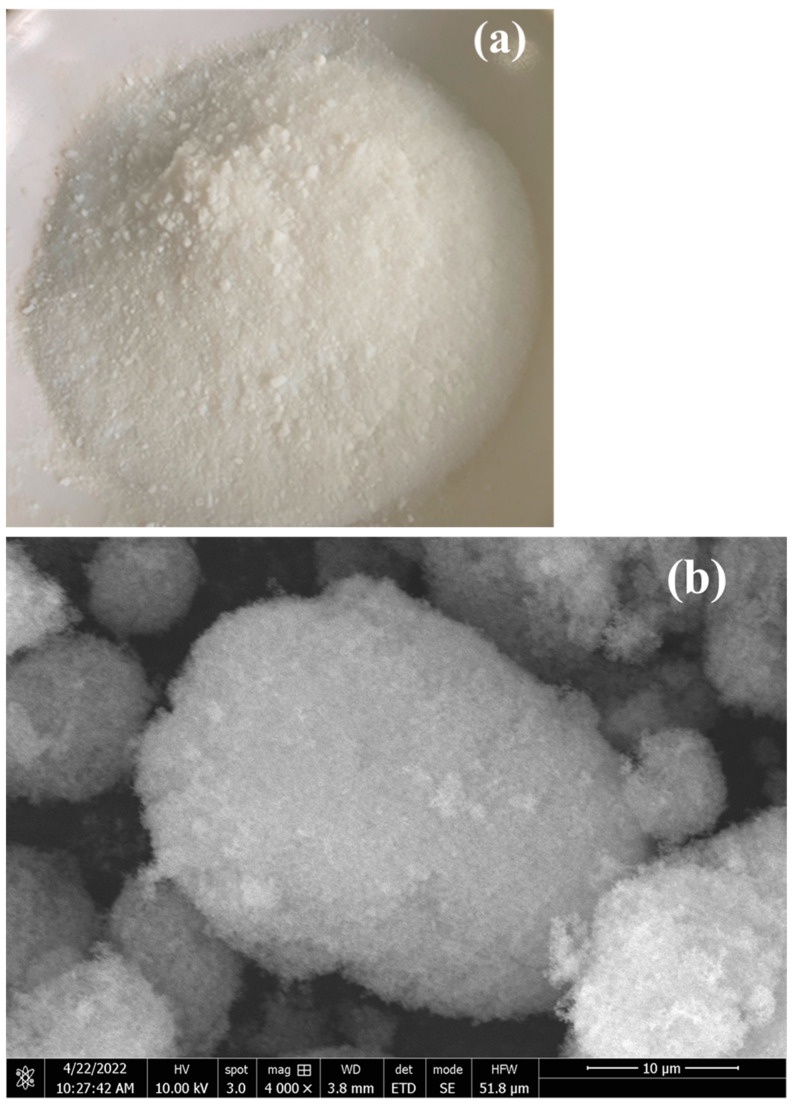
(**a**) Raw material of SiO_2_; (**b**) SEM image of SiO_2_.

In Figure 3a, the raw material of SiO_2_ can be seen. SiO_2_ is a general term that is used for fine powder or ultrafine particles of anhydrous and hydrous silica or silicates. It is commonly known as nano-fumed silica, and is a white, non-toxic, amorphous, mesh-like, and flocculent quasi-silica. Micro-spherical powder with a granular structure is insoluble in water, soluble in caustic alkali solution and hydrofluoric acid, has high temperature resistance, is non-flammable, is odorless, and has good electrical insulation. SiO_2_ is used as a carrier material for the adsorption of PCM, such that the phase transition of PCM occurs inside SiO_2_. The pores inside SiO_2_ and the porous capillary structure firmly adsorb the phase change material, such that the phase change material will not leak. It macroscopically becomes a form of solid-solid phase transition, and a relatively stable structure is formed. The interior of SiO_2_ contains many pores, and the porous capillary structure firmly adsorbs the PCM, such that the phase change material will not leak. Therefore, it macroscopically becomes a form of solid phase transition, and a relatively stable structure is formed. By observing the appearance characteristics of silica with a scanning electron microscope, one can see silica’s surface structure (Figure 3b). The fact that silica is loose and porous, and has a huge specific surface area, ensures that it can adsorb more phase change materials. The amorphous flocculent particle structure allows for the easy observation of its adsorption state.

**Figure 4:**
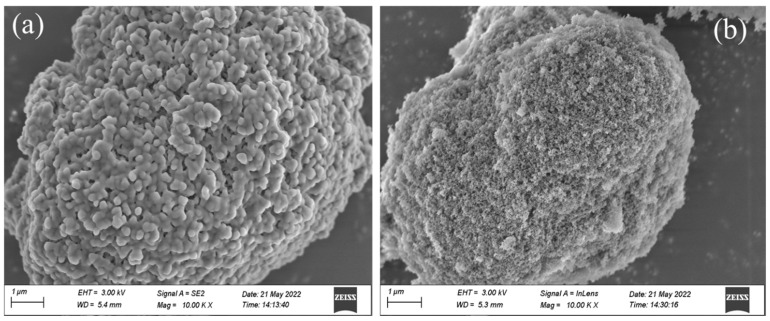
SEM images of composite shaped PCM for (**a**) PEG-400/SiO_2_ and (**b**) Tet/SiO_2_.

The SEM images of different composite shaped PCMs can be seen in Figure 4. During the preparation process, the liquid PEG-400/Tet was mainly adsorbed in the pore structure of SiO_2_ or on the inner and outer surfaces through the combined action of capillary force and surface tension, and the membrane material was then used for encapsulation. From Figure 4a, it can be seen that the capsule wall solution of PEG-400/SiO_2_ composite shaped PCM played an important role in the encapsulation, and SiO_2_ completely encapsulated the phase change material, which had a good appearance, a smooth surface, and uniform particles. Figure 4b shows that the Tet/SiO_2_ composite shaped PCM was agglomerated, the surface of the shaped phase change material was rough, there were many pores on the surface, and no capsules were formed, which indicates the adsorption of PCM into the pores of the carrier, while the surface maintained the porous and loose state of SiO_2_, and the capsule wall solution did not play a good role in the encapsulation. The reason for the appearance of Tet/SiO_2_ composite shaped PCM may be due to the solution in the capsule wall not being enough to wrap the shaped phase change material, or it could be due to there being too much carrier material and too little PCM, which resulted in uneven adsorption.

### 3.2. Thermal Reliability

**Figure 5:**
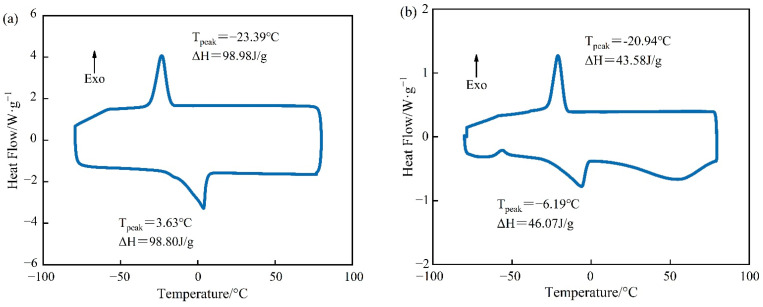
DSC curves of PCM for (**a**) pure PEG-400 and (**b**) PEG-400/SiO_2_ composite shaped PCM.

**Figure 6:**
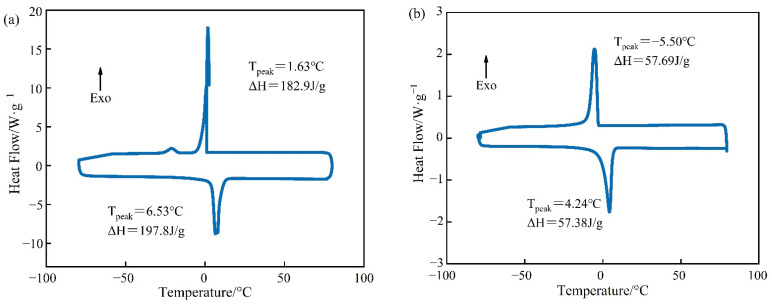
DSC curves of PCM for (**a**) pure Tet and (**b**) Tet/SiO_2_ composite shaped PCM.

The DSC curves of PEG-400/SiO_2_ and Tet/SiO_2_ composite shaped PCMs can be seen in Figure 5 and Figure 6. Figure 5 shows that pure PEG-400 only had one endothermic peak and one exothermic peak, which indicates that the PEG-400 material was relatively pure and without impurities. During the endothermic process of pure PEG-400, the melting peak temperature was 3.63 °C and the melting enthalpy was 98.80 J/g. In the exothermic process, the solidification peak temperature of PEG-400 was −23.39 °C and the solidification enthalpy was 98.98 J/g. The theoretical melting enthalpy and solidification enthalpy of PEG-400/SiO_2_ composite shaped PCM were calculated using Equation (1), and they were 83.98 J/g and 84.13 J/g, respectively. Figure 5 shows that the melting peak temperature of PEG-400/SiO_2_ composite shaped PCM was −6.19 °C, the solidification peak temperature was −20.94 °C, the melting enthalpy was 43.58 J/g, and the solidification enthalpy was 46.07 J/g. These were 51.89% and 54.76% of their theoretical values, which indicates that SiO_2_ has a certain influence on the phase transition behavior of PEG-400 in the composite phase transition system. In addition, Figure 5 shows that the PEG-400/SiO_2_ composite shaped PCM had an additional peak in the endothermic process, which was potentially due to there being too much wall material. The large pore structure of SiO_2_ exhibits a strong adsorption and immobilization effect on PEG-400 molecules, restricting the free movement of PEG-400 molecular chains during the phase transition process, which results in a decrease in the phase transition heat storage density of the composite phase transition system. This effect can be beneficial for preventing liquid phase PEG-400 leakage and improving the shaping effect of the composite phase change material.

Figure 6 demonstrates that both pure Tet and Tet/SiO_2_ composite shaped PCM had only one endothermic peak and one exothermic peak, which indicates that both the pure Tet and Tet/SiO_2_ composite shaped PCM were relatively pure and without impurities. In the endothermic process of pure Tet, the melting peak temperature was 6.53 °C and the melting enthalpy was 197.8 J/g, while in the exothermic process, the solidification peak temperature was 1.63 °C and the solidification enthalpy was 182.9 J/g. The theoretical melting enthalpy and solidification enthalpy of Tet/SiO_2_ composite shaped PCM were calculated using Equation (1), and they were 168.1 J/g and 155.5 J/g, respectively. Figure 6 also shows that the melting peak temperature of Tet/SiO_2_ composite shaped PCM was 4.24 °C and the solidification peak temperature was −5.50 °C, while the melting enthalpy was 57.38 J/g and the solidification enthalpy was 57.69 J/g, which were their theoretical values of 34.13% and 37.10%. This may be because, in the preparation process of Tet/SiO_2_ composite shaped PCM, the core material of the capsule is composed of 50% Tet and 50% SiO_2_, whereas SiO_2_ does not have the function of heat storage. Following the drying and shaping of microencapsulated shaped PCM, the capsule wall material makes up part of the total mass, and the capsule wall material does not possess a heat storage function. Therefore, the phase transformation enthalpy of the prepared composite shaped PCM is lower than that for Tet. As the bladder wall material has a certain degree of thermal insulation and Tet has a certain degree of supercooling, the premature loss of heat can be avoided, such that the phase transition point and phase transition peak of the composite shaped PCM have greater suitability for road surface heating in winter.

### 3.3. Thermal Stability

**Figure 7:**
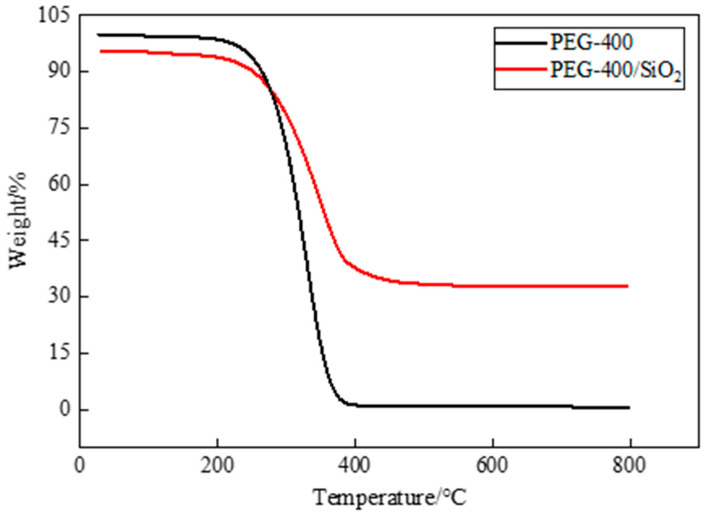
TGA curves of PEG-400 and PEG-400/ SiO_2_ composite shaped PCM.

**Figure 8:**
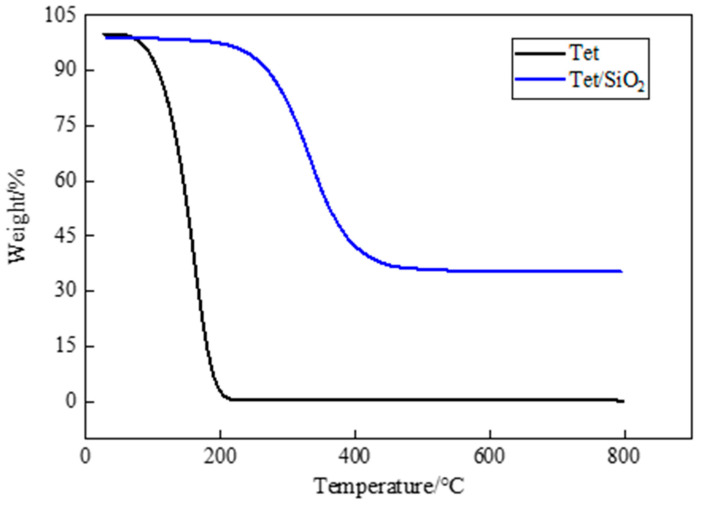
TGA curves of Tet and Tet/ SiO_2_ composite shaped PCM.

Thermal stability is an important factor in PCM research and application, and thermogravimetric analysis is a widely used means for the evaluation of the thermal stability of PCM. TGA is a testing technique in which the mass of the test sample changes as the temperature changes. If the tested sample reacts at a certain temperature, the change that is recorded by the thermogravimetric analysis curve can be used to analyze the relationship between the mass and temperature, which is determined by the mass change and the degree to which a material reacts. In Figure 7, the TGA test results of pure PEG-400 and PEG-400/SiO_2_ composite shaped PCM can be seen, and in Figure 8, the TGA test results of pure Tet and Tet/SiO_2_ composite shaped PCM are displayed. The test temperature range of the PCMs was 30~800 °C. All of the tests were conducted in a nitrogen atmosphere, and all of the PCMs were weightless in one step.

Figure 7 shows that pure PEG-400 was almost completely lost at high temperatures and could not be used directly. The prepared PEG-400/SiO_2_ composite shaped PCM could be used in a high-temperature environment. During the initial heating stage, the mass loss of PEG-400/SiO_2_ composite shaped PCM was not particularly obvious. When the temperature exceeded the initial weight loss temperature of 247.31 °C (at 5% weight loss), PEG started to thermally decompose rapidly between 250 °C and 400 °C. The mass gradually decreased, and the weight loss rate was approximately 93.46%, which was potentially a result of the decomposition of PEG molecules. When the temperature continued to increase, the thermal decomposition of the composite shaped PCM stopped, and the final residual mass was approximately 32.81%, which included the mass of SiO_2_ and the residue of the capsule wall. In the TGA test temperature range, the mass of SiO_2_ did not decrease, and its physical and chemical properties both remained stable.

From Figure 8, it can be seen that pure Tet was almost completely decomposed at high temperatures, and could not be used directly. Figure 8 also shows that, as is consistent with the PEG-400/SiO_2_ composite shaped PCM, the mass loss of the Tet/SiO_2_ composite shaped PCM in the initial heating stage was particularly small. When the temperature exceeded the initial weight loss temperature of 246.72 °C (at 5% weight loss), the Tet molecule started to decompose rapidly, the sample mass gradually decreased, and the weight loss rate became 93.98%.

### 3.4. Properties of the Recycled Aggregate Pavement Brick with Composite Shaped PCM

#### 3.4.1. Mechanical Properties

**Figure 9:**
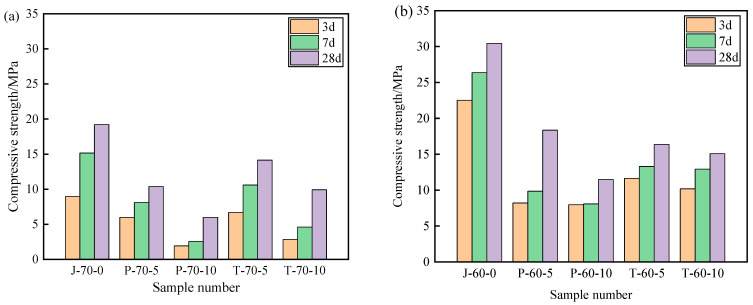
Compressive strength of recycled aggregate pavement bricks containing PEG-400/SiO_2_ and Tet/SiO_2_ composite shaped PCM: (**a**) 70% brick slag; (**b**) 60% brick slag.

**Figure 10:**
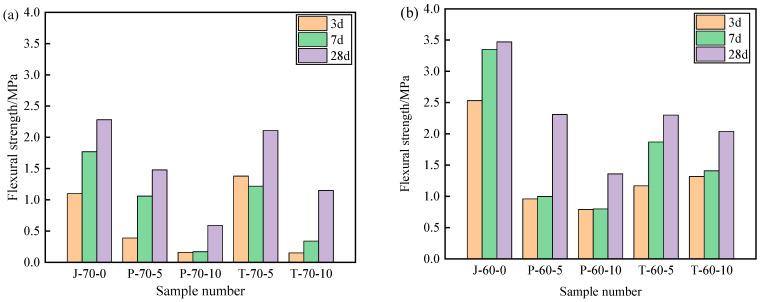
Flexural strength of recycled aggregate pavement bricks containing PEG-400/SiO_2_ and Tet/SiO_2_ composite shaped PCM: (**a**) 70% brick slag; (**b**) 60% brick slag.

Figure 9 and Figure 10 show the compressive strength and flexural strength of the recycled aggregate pavement bricks (40 mm × 40 mm× 160 mm) that contain PEG-400/SiO_2_ composite shaped PCM and Tet/SiO_2_ composite shaped PCM with different recycled aggregate contents. It can be seen that the compressive strength and flexural strength of composite shaped PCM recycled aggregate paving bricks were significantly affected by the amount of recycled aggregate and composite shaped PCM.

Figure 9 and Figure 10 show that, with different percentages of recycled aggregate content, the compressive strength and flexural strength of the obtained composite shaped PCM recycled aggregate paving bricks all exhibited similar trends after 3, 7, and 28 days. Specifically, regarding the PEG-400/SiO_2_ composite shaped PCM recycled aggregate paving bricks mixed with 60% and 70% recycled aggregate, the compressive strengths of P-60-5 specimens mixed with 60% recycled aggregate and 5% composite shaped PCM were 8.21 MPa, 9.85 MPa, and 18.35 MPa after 3, 7, and 28 days. In contrast, the compressive strength of the P-70-5 specimen with 70% recycled aggregate was found to be significantly lower than that of P-60-5, with values of 5.98 MPa, 8.11 MPa, and 10.37 MPa. In addition, the compressive strength and flexural strength of the P-60-5 specimens were higher than those of the P-60-10 specimens at 3, 7, and 28 days, which indicates that the recycled aggregate and PEG-400/SiO_2_ composite shaped PCM had a significant influence on the mechanical properties of composite shaped PCM recycled aggregate pavement bricks, and that a greater content of recycled aggregate and composite shaped PCM leads to a sharper decline in the mechanical properties of composite shaped PCM recycled aggregate pavement bricks. Similar results were also obtained for the new PEG-400/SiO_2_ composite PCM concrete block that was prepared by Xu et al. [26]. They discovered that the amount of PEG-400/SiO_2_ composite PCM negatively impacted the compressive strength of concrete. Quan et al. [34] found that adding PEG phase change material reduces the strength, but the use of PEG phase change material in the form of filler or fine aggregate for engineering construction has no significant effect on the mechanical properties of building materials.

Regarding the Tet/SiO_2_ composite shaped PCM recycled aggregate paving bricks mixed with 60% and 70% recycled aggregate, a similar weakened mechanical property phenomenon as was seen with the PEG-400/SiO_2_ composite shaped PCM recycled aggregate paving bricks was observed. For example, the compressive strengths of T-60-5 specimens that were mixed with 60% recycled aggregate and 5% composite shaped PCM were 11.63 MPa, 13.29 MPa, and 16.36 MPa after 3, 7, and 28 days, while the flexural strengths were 1.17 MPa, 1.87 MPa, and 2.30 MPa at the same points. Ensuring that the content of 60% recycled aggregate remained unchanged when Tet/SiO_2_ composite shaped PCM increased from 5% to 10%, the compressive strength and flexural strength of the Tet/SiO_2_ composite shaped PCM recycled aggregate paving bricks decreased by approximately 0.6–1.5 MPa and 0.2–0.5 MPa. In addition, Figure 8 and Figure 9 show that the compressive strength and flexural strength of the T-70-5 and T-70-10 Tet/SiO_2_ composite shaped PCM recycled aggregate pavement bricks with 70% recycled aggregate were lower than those of the Tet/SiO_2_ composite shaped PCM recycled aggregate pavement bricks with 60% recycled aggregate at 3, 7, and 28 days. As is consistent with the aforementioned trend, the larger the proportion of recycled aggregate and composite shaped PCMs incorporated is, the lower the mechanical properties of the composite shaped PCM recycled aggregate pavement bricks will be.

Several mechanisms can be used to explain this strength reduction of the two composite shaped PCM recycled aggregate paving bricks: (1) The material structure of the recycled aggregate is loose, many internal defects exist, and its strength is low [2]. (2) The high water absorption rate of broken brick aggregate can significantly affect the hydration of the cement inside the recycled brick, resulting in a negative impact on the overall strength of the brick [2]. (3) The addition of composite shaped PCM particles increases the size of the voids inside the recycled aggregate bricks, and as the added ratio of composite shaped PCM increases, the internal structure of the prepared composite shaped PCM recycled aggregate pavement bricks becomes less tightly combined, inevitably resulting in a decrease in overall strength [35]. Generally, the incorporation of PEG-400/SiO_2_ and Tet/SiO_2_ composite shaped PCM reduces the strength of composite shaped PCM recycled aggregate pavement bricks. The strength of the Tet/SiO_2_ composite shaped PCM recycled aggregate pavement brick is significantly greater than that of the PEG-400/SiO_2_ composite shaped PCM recycled aggregate pavement brick, and its strength at 28 days satisfies the national MU15 strength grade requirement.

#### 3.4.2. Thermal Conductivity

**Figure 11:**
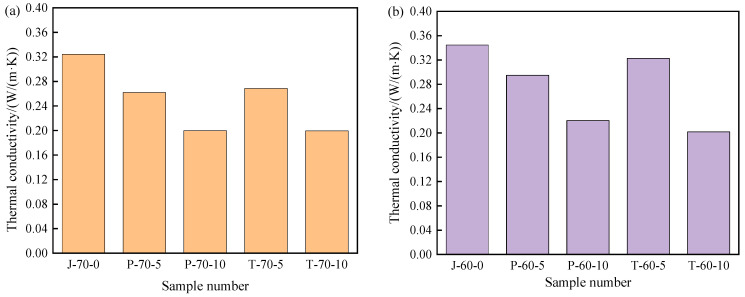
Thermal conductivity of recycled aggregate pavement bricks containing PEG-400/SiO_2_ and Tet/SiO_2_ composite shaped PCM: (**a**) 70% brick slag; (**b**) 60% brick slag.

The thermal conductivity of composite shaped PCM recycled aggregate pavement brick is of great importance in the composite shaped PCM heat exchange rate. Therefore, in order to effectively study the effect that the addition of composite shaped PCM and different amounts of recycled aggregate has on the thermal conductivity of composite shaped PCM recycled aggregate pavement brick, thermal conductivity measurements were taken. The results can be seen in Figure 11.

The addition of composite shaped PCM obviously reduced the thermal conductivity of the recycled aggregate pavement bricks. From Figure 11, it can be seen that as the content of PEG-400/SiO_2_ composite shaped PCM and Tet/SiO_2_ composite shaped PCM increased, the content of recycled aggregate increased, and the thermal conductivity of composite shaped PCM recycled aggregate pavement bricks exhibited a decreasing trend. Figure 11a shows that when the recycled aggregate content was 70%, the thermal conductivity of ordinary recycled aggregate pavement brick was 0.324 W/(m·K). When the PEG-400/SiO_2_ composite shaped PCM contents were 5% and 10%, the thermal conductivities of the composite shaped PCM recycled aggregate pavement brick were 0.262 W/(m·K) and 0.200 W/(m·K). In comparison to ordinary recycled aggregate pavement bricks, these were reduced by 19.1% and 38.3%, respectively. When the Tet/SiO_2_ composite shaped PCM content was 5% and 10%, the thermal conductivity of the composite shaped PCM recycled aggregate pavement brick was 0.267 W/(m·K) and 0.199 W/(m·K), 17.6% and 38.6% lower than that of ordinary recycled aggregate pavement bricks.

Figure 11b shows that when the recycled aggregate content was 60%, the thermal conductivity of the ordinary recycled aggregate pavement brick was 0.345 W/(m·K), while when the peg-400/SiO_2_ composite shaped PCM content was 5% and 10%, the thermal conductivity of the composite shaped PCM recycled aggregate pavement brick was 0.295 W/(m·K) and 0.220 W/(m·K), 14.5% and 36.2% lower than that of ordinary recycled aggregate pavement bricks. When the Tet/SiO_2_ composite shaped PCM content was 5% and 10%, the thermal conductivity of the composite shaped PCM recycled aggregate pavement brick was 0.323 W/(m·K) and 0.202 W/(m·K), 6.4% and 41.4% lower than that of ordinary recycled aggregate pavement bricks. The addition of composite shaped PCM affects the thermal conductivity of the composite shaped PCM recycled aggregate pavement brick, which is potentially due to the existence of composite shaped PCM. The pores in the composite shaped PCM recycled aggregate pavement brick increase, thereby increasing the air content in the specimen; this ultimately affects the heat flow transmission in the composite shaped PCM recycled aggregate pavement brick and decreases the thermal conductivity.

#### 3.4.3. Analysis of the Temperature Control Effect

**Figure 12:**
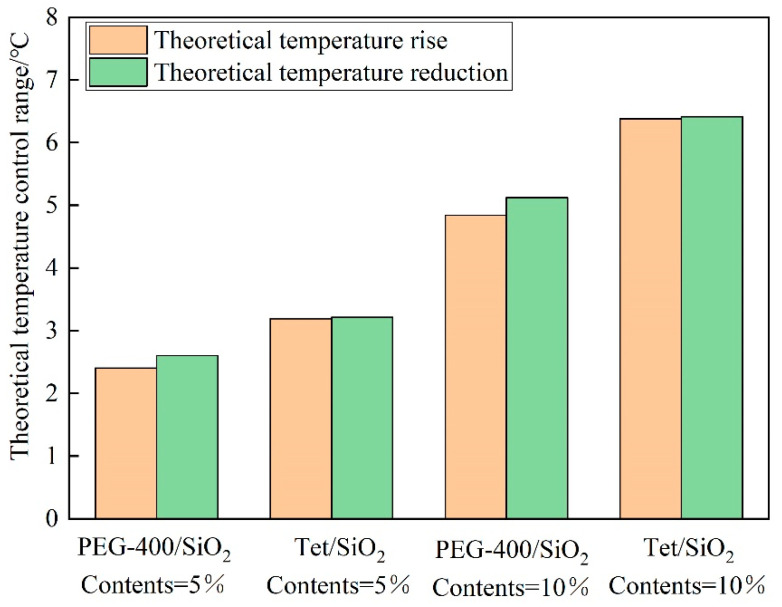
Theoretical temperature control range of PEG-400/SiO_2_ and Tet/SiO_2_ composite shaped PCM recycled aggregate pavement bricks.

Because the thermal conductivity cannot directly reflect the heat storage capacity of the composite shape-setting phase change material recycled pavement bricks, the temperature control effect of the prepared pavement bricks can be assessed by computing the theoretical temperature control range. Figure 12 illustrates the theoretical temperature control range of the two-phase change materials at various dosages. As can be seen, the temperature control range of various phase change materials under different dosages is in the range of 2.4 to 6.4 °C. Because the melting enthalpy of the two-phase change materials is lower in comparison to the solidification enthalpy, the theoretical cooling range of the recycled pavement bricks of the phase change material is smaller than the theoretical heating range. When the dosage of the phase change material is the same, the theoretical temperature control range of Tet/SiO_2_ composite shaped PCM recycled pavement bricks is greater in contrast to the PEG-400/SiO2 composite shaped PCM recycled pavement bricks. This points to the fact that the temperature control effect of Tet/SiO_2_ composite-shaped PCM is better. The theoretical temperature control range for the same phase change material increases with the increasing doping amount. It can be seen that the doping amount and the enthalpy value of the composite phase change material are the key factors determining the temperature control effect.

## 4. Conclusions

In this paper, two novel composite-shaped PCMs—PEG-400/SiO_2_ and Tet/SiO_2_—were prepared, and their microstructures and thermal properties were characterized. The results of the research demonstrate that the adsorption of PEG-400 to SiO_2_ is stronger than that of Tet to SiO_2_, and the shaping effect of PEG-400/SiO_2_ is better than that of Tet/SiO_2_. The DSC analysis curve reveals that the melting peak temperature and the solidification peak temperature of PEG-400/SiO_2_ composite-shaped PCM are −6.19 °C and −20.94 °C, respectively, whereas the melting enthalpy and solidification enthalpy are 43.58 J/g and 46.07 J/g, respectively. Tet/SiO_2_ composite-shaped PCM has melting and solidification peak temperatures of 4.24 °C and −5.50 °C, respectively. The melting enthalpy and solidification enthalpy are 57.38 J/g and 57.69 J/g, respectively. According to the TGA curves, both phase change materials have good thermal stability. Recycled aggregate pavement bricks were created using two-phase change materials. The analysis of their mechanical properties and thermal conductivity highlighted that the incorporation of PEG-400/SiO_2_ and Tet/SiO_2_ composite-shaped PCM would weaken the compressive strength and flexural strength of recycled aggregate pavement bricks. In comparison to PEG-400/SiO_2_ composite shaped PCM recycled aggregate pavement brick, the strength of Tet/SiO_2_ composite shaped PCM recycled aggregate pavement brick is evidently higher. The strength of P-60-5 recycled aggregate paving bricks is greater than that of P-70-10 recycled aggregate paving bricks. Tet/SiO_2_ composite-shaped PCM has better thermal conductivity than PEG-400/SiO_2_. The temperature control effect of Tet/SiO_2_ composite shaped PCM recycled pavement brick is better than that of PEG-400/SiO_2_ composite shaped PCM recycled pavement brick. The theoretical temperature control range increases when the amount of phase change material increases. To summarize, the temperature control effect and mechanical properties of Tet/SiO_2_ composite stereotyped PCM pavement bricks are better than PEG-400/SiO_2_ composite stereotyped PCM pavement bricks. However, due to the increased price of Tet/SiO_2_ composite-shaped PCM materials, the economic cost issue must be taken into account in practical applications.

## Figures and Tables

**Table 1:** Physical parameters of the capsule core and the capsule wall materials.

Material Types	Parameters	Purity Grade	Characteristics	Average Molecular Weight
Capsule core material	PEG-400	Analytical pure	Colorless oily liquid	380–420
	Tet	Analytical pure	Colorless liquid	198.44
Capsule wall material	Anhydrous alcohol	Analytical pure	Colorless liquid	40.07
	Diisooctyl sebacate	Chemically pure	Colorless oily liquid	426.67
	Ethyl cellulose	Analytical pure	White powdery solid	448.47

**Table 2:** Proportions of the composite shaped PCM.

Composite Shaped PCM	Ratio of PCM to SiO_2_	Ratio of Composite Shaped PCM to Liquid Phase Gel System	Mass Fraction of Gel System
PEG-400/SiO_2_	5:3	1:0.45	8.5%
Tet/SiO_2_	1:1	1:0.45	8.5%

**Table 3:** Mix proportion of composite shaped PCM recycled aggregate pavement bricks (size 40 mm × 40 mm × 160 mm).

Sample Number	Recycled Aggregate		Composite Shaped PCM		Cement + Water		Water/Cement
	Mass (g)	Ratio (%)	Mass (g)	Ratio (%)	Mass (g)	Ratio (%)	
J-70-0	945	70	0	0	405	30	0.5
J-60-0	810	60	0	0	540	40	0.5
P-70-5	945	70	67.5	5	337.5	25	0.5
P-70-10	945	70	135	10	270	20	0.5
P-60-5	810	60	67.5	5	472.5	35	0.5
P-60-10	810	60	135	10	405	30	0.5
T-70-5	945	70	67.5	5	337.5	25	0.5
T-70-10	945	70	135	10	270	20	0.5
T-60-5	810	60	67.5	5	472.5	35	0.5
T-60-10	810	60	135	10	405	30	0.5

**Table 4:** Grading of recycled aggregate pavement bricks.

Strength Grading	Average Compressive Strength/MPa	Minimum Value of Single Brick/MPa
MU20	20.0	16.0
MU15	15.0	12.0
MU10	10.0	8.0
MU7.5	7.5	6.0
MU5	5.0	4.0
MU3.5	3.5	2.8

## Data Availability

The general data are included in the article. Additional data are available on request.
